# Tyrphostin AG17 inhibits adipocyte differentiation in vivo and in vitro

**DOI:** 10.1186/s12944-018-0784-7

**Published:** 2018-05-29

**Authors:** Alberto Camacho, Juan Carlos Segoviano-Ramírez, Adriana Sánchez-Garcia, Jose de Jesus Herrera-de la Rosa, Jaime García-Juarez, Carlos Alberto Hernandez-Puente, Geovana Calvo-Anguiano, Sergio Rodolfo Maltos-Uro, Alejandra Olguin, Gabriel Gojon-Romanillos, Gabriel Gojon-Zorrilla, Rocio Ortiz-Lopez

**Affiliations:** 1 Departamento de Bioquímica y Medicina Molecular, Facultad de Medicina, Universidad Autónoma de Nuevo León (UANL), Monterrey, Mexico; 2 Unidad de Neurometabolismo, Centro de Investigación y Desarrollo en Ciencias de la Salud (CIDICS), UANL, Monterrey, Mexico; 3 Departamento de Histologia, UANL Facultad de Medicina, Monterrey, Mexico; 4 Unidad de Bioimagen, UANL, CIDICS, Monterrey, Mexico; 5 Unidad de Genómica, UANL, CIDICS, Monterrey, Mexico; 6 Unidad de Modelos Experimentales, UANL, CIDICS, Monterrey, Mexico; 7 Unidad de Innovación Biomédica, A.C, Monterrey, Nuevo León Mexico; 8 Dirección de Innovación Disruptiva, Ayon Industries, Monterrey, Mexico; 9 Dirección de Investigación y Desarrollo, Ayon Industries, Monterrey, Mexico; 10 División de Ciencias de la Salud, Instituto Tecnológico y de Estudios Superiores de Monterrey (ITESM), Monterrey, NL Mexico; 11 Unidad de Genómica. Unidad de Neurometabolismo, CIDICS, UANL, Dr Carlos Canseco s/n. Colonia Mitras Centro, CP64460 Monterrey, Nuevo León Mexico

**Keywords:** Tyrphostin, AG17, Adipogenesis, Obesity, Hepatic steatosis, Oxidative phosphorylation, Thermogenesis, Mitochondrial uncoupling, Adipocyte differentiation

## Abstract

**Background:**

Excessive subcutaneous adiposity in obesity is associated to positive white adipocyte tissue (WAT) differentiation (adipogenesis) and WAT expandability. Here, we hypothesized that supplementation with the insulin inhibitor and mitochondrial uncoupler, Tyrphostin (T-AG17), in vitro and in vivo inhibits adipogenesis and adipocyte hypertrophy.

**Methods:**

We used a 3T3-L1 proadipocyte cell line to identify the potential effect of T-AG17 on adipocyte differentiation and fat accumulation in vitro. We evaluated the safety of T-AG17 and its effects on physiological and molecular metabolic parameters including hormonal profile, glucose levels, adipogenesis and adipocyte hypertrophy in a diet-induced obesity model using C57BL/6 mice.

**Results:**

We found that T-AG17 is effective in preventing adipogenesis and lipid synthesis in the 3T3-L1 cell line, as evidenced by a significant decrease in oil red staining (*p* < 0.05). In obese C57BL/6 mice, oral administration of T-AG17 (0.175 mg/kg for 2 weeks) lead to decreased fat accumulation and WAT hypertrophy. Further, T-AG17 induced adipocyte apoptosis by activating caspase-3. In the hepatocytes of obese mice, T-AG17 promoted an increase in the size of lipid inclusions, which was accompanied by glycogen accumulation. T-AG17 did not alter serum biochemistry, including glucose, insulin, leptin, free fatty acids, creatinine, and aspartate aminotransferase.

**Conclusion:**

T-AG17 promotes adipocyte apoptosis in vivo and is an effective modulator of adipocyte differentiation and WAT hypertrophy in vitro and in vivo. Therefore, T-AG17 may be useful as a pharmacological obesity treatment.

## Background

Excessive subcutaneous adiposity and its accumulation into visceral depot during obesity are major risk factors for developing type 2 diabetes (T2DM) and several other chronic metabolic disorders [1, 2]. Therefore, identifying therapeutic targets to treat the metabolic failures associated with obesity could reduce or prevent the development of these incurable metabolic disorders. Two cellular signaling pathways that may contain potential drug targets are the white adipose tissue (WAT) differentiation pathway, also referred to as adipogenesis, and the WAT expandability pathway.

During early development, mesenchymal stem cells differentiate into chondrocytes, osteoblasts, myoblasts and adipocytes [3]. Adipocyte differentiation from mesenchymal stem cells is modulated by signaling cascades involving bone morphogenetic protein-4 and peroxisome proliferator-activated receptor (PPAR) β/δ, which support the gene expression of PPARγ [4]. In adults, ectopic accumulation of adipocytes might be caused by dysfunction in differentiation pathways, which creates an inability to induce differentiation of adipocyte precursor cells [3, 5] or induce adipocyte de-differentiation [6]. On the other hand, once obesity is reached, the number of new adipocytes decreases, and adipocytes become hypertrophic, reaching their expandability limit until fat accumulates around ectopic organs, resulting in metabolic complications [7, 8].

Drugs that act on mitochondria have been used to combat fat accumulation by forcing cells to use stored energy. Mitochondrial oxphos uncouplers create a futile cycle of glyceride and fatty acid oxidation without generating adenosine triphosphate (ATP). 2,4-dinitrophenol (DNP) is one of the best known uncoupler of oxphos widely used as a weight loss agent between 1933 and 1938; however, DNP has been banned due to its high acute toxicity [9]. Further studies on DNP and other related prodrugs consistently show that the toxic effects are dose-dependent. Mild mitochondrial uncoupling (MMU) with low doses, which seem effective at thermoneutrality (30 °C) conditions, may provide a promising strategy to reduce body weight with general tolerability [10–18]. Side effects of DNP exposure might be prevented, and MMU might also promote longevity through decreased ROS levels, mitochondrial biogenesis, downregulation of the mTOR and insulin signaling pathways, and upregulation of autophagy [12, 13]. Of interest, these effects are similar to those seen in caloric restriction [12, 15, 19] and are consistent with the “uncoupling to survive” hypothesis [20]. These potentially beneficial effects of MMU may also be induced by niclosamide ethanolamine [21], salsalate [22], TTFB [23], CZ5 [24], FCCP [6, 17, 25–27], the niclosamide-ethanolamine aduct [21] and certain tyrphostins [28].

Tyrphostins belong to the benzylidenemalononitrile family, which possess a benzene ring pharmacophore, an exocyclic carbon-carbon double bond, and a cyano group (CN) located at the same side of the molecule (cis) as the aromatic ring [29, 30]. The tyrphostin T-AG17 is a highly selective, reversible inhibitor of epidermal growth factor receptor-induced phosphorylation of tyrosine residues of intracellular proteins [31], and its cellular effect is dose-dependent growth inhibition. T-AG17 has potential therapeutic value for treating neurodegenerative disorders [28], atherosclerosis [32], dyslipidemia [33], restenosis [34], and cancer/cell hyperproliferation [35], partially due to the reduction of free radical production in mitochondria [28, 36], activation of Nrf2 transcription factor [37], reduction of CDK2 kinase activity, as well as causing reduced p21 and p16 protein levels [38] and decreased in STAT3 phosphorylation [39]. Notably, T-AG17 suppresses insulin-mediated fatty acid synthesis in WAT of rats [33], and tyrphostins B46 and A47 (which bear the same pharmacophore as T-AG17) block GLUT1- mediated intracellular glucose transport [40]. Importantly, T-AG17 is also a potent inhibitor of mitochondrial oxphos and is capable of increasing energy expenditure. However, the effects of T-AG17 on adipocyte differentiation, adipose tissue hypertrophy and body organ toxicity have not been evaluated.

Considering the significant suppressive effects of T-AG17 on insulin signaling, we hypothesized that T-AG17 might promote inhibition of adipogenesis and/or adipocyte hypertrophy. Specifically, we seek to determine if T-AG17: 1) decreases lipid accumulation in the 3T3-L1 adipocyte cell line induced by insulin and 2) promotes adipocyte apoptosis in a diet-induced obesity mouse model.

## Methods

### Reagents and antibodies

3T3-L1 preadipocyte cell line (Cat. CL-173) and newborn calf serum (Cat. 30-2030) were purchased from ATCC, Inc. Dulbecco’s Modified Eagle’s Medium-high glucose (Caisson Labs, Cat.), Penicillin-Streptomycin (Cat. P4333), Fetal Bovine Serum (Gibco), Oil Red O (Cat. O0625), Dimethyl sulfoxide (Cat. D2650), isopropyl alcohol (Cat. W292907), Formalin solution neutral buffered 10% (Cat. HT501128), Ethyl alcohol (Cat. E7023), Harris hematoxylin (Cat. HHS16), Sodium citrate (Cat. 1613859), Triton X-100 (Cat. X100), Corning® cell culture flasks surface area 75 cm2, canted neck, cap (vented) (CLS430641) were from SIGMA-ALDRICH. Paraplast® Embedding Media, (Cat. 15159-409, McCormick Scientific), Rabbit polyclonal to Active + pro Caspase 3 (Cat. AB13847, ABCAM), Goat polyclonal secondary antibody to Rabbit IgG H&L (alexa Fluor® 488) preadsorbed (Cat. AB150081, ABCAM), and VECTASHIELD Hardset antifade mounting medium with DAPI (cat no. H-1500 Vector laboratories).

Adipogenesis Assay Kit for 3T3-L1 preadypocyte differentiation was purchased from Abcam (Cat. Ab133102). Also, the primary antibody cleaved Caspase-3 (Asp175) (Cat. 9661. Cell signaling) and secondary antibody Anti-rabbit IgG (H + L), Alexa Fluor® 488 Conjugate (Cat. 4412. Cell signaling) were used. ELISA kits: creatinine (SIGMA. MAK080), aspartate aminotransferase (SIGMA. MAK055), free fatty acids (Roche, 11383175001), insulin (Millipore, Cat. EZRMI-13 k) and leptin (Millipore, Cat. EZML-82 K). Acucheck (Cat. 05987270) and glucose strips (6454011023, Roche). 10% Phosphate-buffered saline (PBS) Formalin solution (Cat. SF100–20, Fischer Scientific), Isopropyl alcohol (Cat. 9084-03 J.T. Baker), Ethyl alcohol (Cat. E7023), Trichloromethane (Cat. 616778, Sigma Aldrich), Acetic acid, glacial (Cat.193829 MP Biomedicals, Inc.), Histological grade xylene (Cat. 534056 Sigma Aldrich), Paraplast® Embedding Media, (Cat. 15159-409, McCormick Scientific), Eosin–Y 7111 (Richard-Allan Scientific), Hematoxylin 7212; (Richard-Allan Scientific), Tissue tek O.C.T. (Cat.4530 Sakura), Oil red (Cat.00625-25g Sigma Aldrich), Schiff’s reagent (Cat. 3952016 Sigma Aldrich), 99% Periodic Acid (Cat. P7875, Sigma Aldrich).

### T-AG17stock solution

The T-AG17 was provided by Ayon Industries (Monterrey, México). T-AG17 was synthesized by reacting (under reflux) 4-hydroxy-3,5-di-tert-butylbenzaldehyde (99.1% pure, purchased from Yongyi Chemicals Group Co., Ltd., Changzhou, Jiangsu, China) with malononitrile in anhydrous ethanol solvent, using ammonium acetate as catalyst. This procedure gives a 95% yield of a light-yellow microcrystalline solid that melts at 141–142 °C, presents UV absorption maxima at 247 and 365 nm and has an Rf value of 0.65 (using benzene as eluent and Merck’s TLC silica gel 60 F_254_ plastic-backed sheets), with only one spot being observed.

The mixed melting point of this compound and authentic T-AG17 (acquired from Cayman Chemical) was 141–142 °C, and its spectroscopic and chromatographic properties were identical to those of the authentic product. This compound is stable, with a shelf life of over 2 years at 25–35 °C. If necessary it may be recrystallized from ethanol.

Stock solutions were prepared in Dimethyl sulfoxide (DMSO; Sigma-Aldrich, D2650). DMSO was used as the vehicle control.

### 3T3-L1 cell line maintenance and treatments

The 3T3-L1 preadipocyte cell line was expanded in Corning® T75 cm^2^ flasks with Dulbecco’s modified Eagle’s medium (DMEM, high glucose 4.5 g/l; (Caisson Labs), supplemented with 10% (vol/vol) newborn calf serum, 50 units/ml penicillin, and 50 μg/ml streptomycin in 5% CO_2_ incubator at 37 °C. After confluence, cells were induced to adipocyte differentiation for 7 days by using DMEM supplemented with 10% vol/vol fetal bovine serum, 1 μM dexamethasone, 0.5 mM isobutylmethylxanthine, 100 nM insulin, and 50 units/ml penicillin and 50 μg/ml streptomycin. The T-AG17 (1 μM) or equivalent concentration of DMSO (vehicle control) was added before (day 0) or after (day 7) adipogenic induction.

### Quantification of lipid accumulation in cells

Effect of T-AG17 on the accumulation of cellular lipid droplets was conducted by comparing T-AG17-treated cells to vehicle control-treated cells after 7 days by using the oil red solution to stain the cells following manufacturer’s instructions. Digital images of the cells were taken with a PrimoVert microscope and the AxioCam ERc5s camera (Zeiss). The stain was extracted from the cells using 60% isopropyl alcohol for 1 h (10 ml/flask), and the extract (1 ml) was measured at 510 nm in the iMark Microplate Absorbance Reader (Bio-Rad).

### Animals and housing

All the experiments were performed using 2 month-old male C57BL/6 mice. Animals were handled according to the NIH guide for the care and use of laboratory animals (NIH Publications No. 80–23, revised in 1996), and animal protocols were approved by the Local Animal Care Committee. All the animals were housed individually in Plexiglas cages and maintained at 20–23 °C in a temperature-controlled room with a 12-h light/dark cycle. Water and food was available ad libitum in the home cage.

### Mice long-term feeding and treatments

Animals were housed a week before the experiment as described above. Mice were exposed to either a high-fat diet (HFD, 45% kcal from fat; Research Diets, D12451) or a basic Chow diet (CHOW, 10% kcal from fat; Research Diets, D12450B) for 13 weeks, as described in our previous studies [41, 42].

T-AG17 or vehicle control were administered orally (via gavage) or injected intraperitoneally (i.p.), and body weight, food intake and water consumption were recorded every week. Doses of 1.75, 5.5, 17.5, 28, 40 and 55 mg/kg were administered once to determine the LD_50_, and doses of 0.175, 0.0175 and 0.00175 mg/kg were administered daily for 15 days to determine the therapeutic effects.

### Tissue sample collection and histological analysis

Mice were sacrificed by cervical dislocation and blood samples were collected using syringe cardiac punch (22G diameter). Serum was isolated as described below. Brain, liver, adipose tissue, pancreas, spleen, gonadal tissue and skeletal muscle were collected and fixed as described below and stained for: hematoxylin/Eosin (H/E; Richard-Allan Scientific), oil red, periodic acid–Schiff (PAS) and active pro-caspase 3 immunofluorescence.

### Serum biochemistry

Blood samples were collected in a Microtainer and centrifuged at 5000 rpm × 10 min. We determined serum biochemical composition including glucose levels by glucose strips and insulin, creatinine, aspartate aminotransferase, leptin and free fatty acids were determined by Elisa kits according to manufacturers’ instructions: creatinine, aspartate aminotransferase, free fatty acids, insulin and leptin.

### Hematoxylin and Eosin staining (H&E)

Samples were fixed in 10% formaldehyde in PBS during 24 h, following by automating processing in an automated (Excelsior ES system®, Thermo Scientific. Inc.). Samples were included in paraffin and 4-μm slices were obtained using a microtome (Microm HM355S. Thermo Scientific. Inc.). Finally, samples were stained with H&E.

### Oil red staining for lipid accumulation in tissue samples

In brief, samples were fixed in 10% formaldehyde in PBS during 24 h, included in “tissue tek” (Tissue-Tek® OCT Compound, TEC Pella, Inc) and 9-μm sections were obtained using a cryostat (Microm HM 550®, Thermo Scientific.Inc). Slides were stained in oil red and counterstained with Harris hematoxylin.

### PAS (Periodic Acid Schiff) staining

Samples were fixed in Carnoy’s solution by 2 h, dehydrated with isopropyl alcohol overnight, pre-included in paraffin and cut 4-μm sections were obtained.

For PAS staining samples were reduced in 0.5% periodic acid solution, placed in Schiff reagent and counterstained in Harris hematoxylin.

Staining protocols were implemented systematically using the automated staining equipment, Varistain Gemini ES® (Thermo Scientific Inc.).

### Immunofluorescence staining

Paraffin slides previously fixed in Carnoy’s solution by 2 h, were processed for antigen retrieval system using 10 mM sodium citrate buffer, pH 6.0 using an automated computing assisted Lab Vision™ PT Module (Thermo Fisher Scientific) and blocking of unspecific antigens was performed using 5% normal goat serum in 0.2% Triton X-100 (Sigma-Aldrich, X100) in PBS 1 mM, pH 7.4, for 1 h. Sections were incubated with active pro-caspase 3 primary antibody (1:2500) for 4 h at room temperature and incubated with Alexa Fluor® 488 Conjugate and anti-rabbit secondary antibody (1:500) for 1 h at room temperature in the darkness. Entire protocol was run into automated immunochemistry computing assisted equipment, Lab Vision™ Autostainer 360® (Thermo Fisher Scientific). Sections were mounted in cover slip using synthetic mounting medium with DAPI (Vector Laboratories, H-1500).

### Light microscopy

Histological slides were observed in a bright field microscope (AxioImager Z1®, Zeiss, Inc.) using a 40× objective. Five fields from each slide, using axio vision software (ver. 4.8.2) were acquired. Morphological analysis of slides from visceral fat tissue were performed by stained with Hematoxylin and eosin stain (H&E), included form of adipocytes. Slides stained with PAS were used to search cytoplasmic inclusions reacting with PAS. Morphological analysis of slides from liver stained with H&E included structure of hepatic lobules, the aspect of cytoplasm from hepatocytes, also the form and aspect of nucleus and it’s chromatin. Presence of oil drops were searched in slides stained with red oil and presence of cytoplasmic inclusions of glycogen in slides stained with PAS.

### Confocal microscopy

Active pro-caspase 3-Cy3 stainning in liver and adipose tissue sections were analyzed by confocal microscopy (Axio imager Z1®. Zeiss, Inc.), using an EC Plan-Neofluar (Plan-Neofluar) 40×/1.30 Oil DIC M27; and 488 nm laser. We scanned for immunoreactive cells using 491/551 nm (Exc/Emi) (LSM 710 scanner, Zeiss, Inc) and ZEN software (Zeiss 2009) for acquisition of 8-bit images collected over a 45,000 μm^2^ surface area.

### Statistical analysis

The data presented here was analyzed using the Student t-test or analysis of variance (ANOVA) with post-hoc tests using the program StatView Version 4.5 (Abacus Concepts, Berkeley, California, United States). For immunofluorescent semiquantitative analysis, we used the ANOVA test followed by Kruskal-Wallis one-way test using Number crunched statistical software (NCSS, LLC, Utah, United States). The data are presented as mean ± SEM, unless otherwise stated; *p* < 0.05 was considered significant. The significance levels displayed on figures are as follows: * indicates *p* < 0.05, ** *p* < 0.001.

## Results

### T-AG17 blocks adipocyte differentiation in 3T3-L1 cell line

**Fig. 1:**
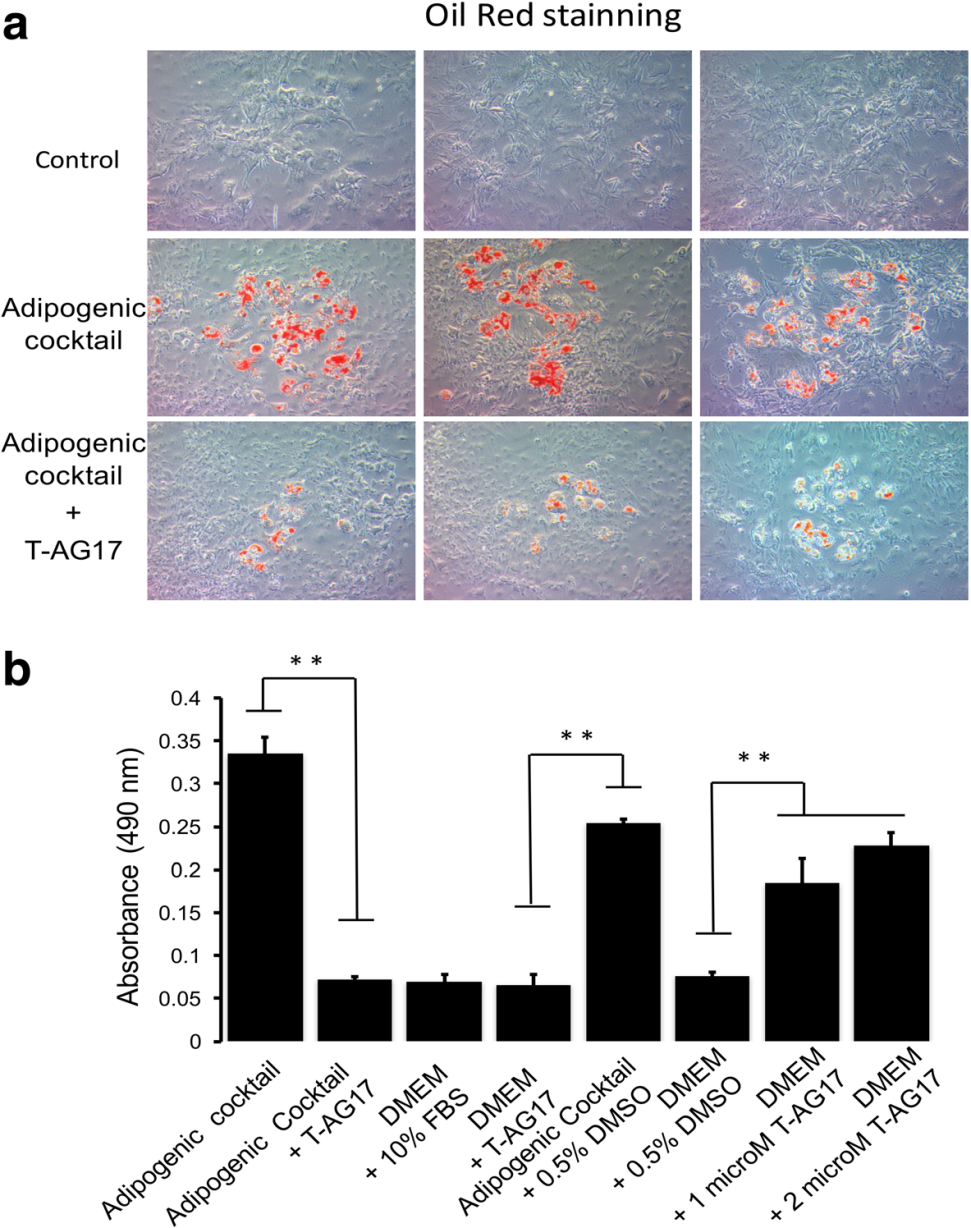
T-AG17 inhibits adipocyte differentiation. **a** 3T3-L1 preadipocyte cells were plated and stimulated with adipogenic cocktail (insulin stimulation). Preadipocytes were incubated with 1μM T-AG17 during 7 days. 3T3-L1 preadypocite lipid droplet staining showed lipid accumulation in cells treated with the adipogenic cocktail. Lipid accumulation was not detected in cells treated with 1μM T-AG17. **b** Adipocyte differentiation was evaluated by quantifying absorbance at 490 nm. Graphs show mean ± SEM for triplicate experiments and statistical significance after using unpaired Student’s t test. *p < 0.05. *n* = 3. In some experiments, T-AG17 was added after preadipocyte differentiation (1 and 2 μM)

We tested whether T-AG17 prevents the adipocyte differentiation of the 3T3-L1 cell line. After cells were confluent, adipocyte differentiation was induced for 7 days in the presence of T-AG17 or vehicle control (DMSO). Adipocyte differentiation was detected by cellular uptake of the oil red stain, which was strikingly induced by treatment with an adipogenic cocktail (insulin stimulation) (Fig. 1a). In the presence of the adipogenic cocktail with T-AG17 (1 μM), there was a visible reduction in the number of positively stained cells and degree of staining per cell (Fig. 1a).

In order to quantify the amount of oil red that the cells absorbed, we extracted the stain and read the absorbance of the extract by a microplate absorbance reader (Fig. 1b). The adipogenic cocktain caused a significant increase in extract absorbance as compared to the DMEM + 10% FBS control treatment group. No differentiation was observed with the DMEM + 10% FBS, DMEM + T-AG17 and DMEM + 0.5% DMSO control treatment groups as expected. T-AG17 treatment on day 0 (when the adipogenic cocktail was first added) caused a significant decrease in absorbance to levels similar to the DMEM + 10% FBS control levels. We found a modest decrease with the vehicle control treatment (adipogenic cocktail + 0.05% DMSO). This finding suggested that T-AG17 prevents adipocyte differentiation.

### Acute doses of T-AG17 and determination of LD_50_

**Fig. 2:**
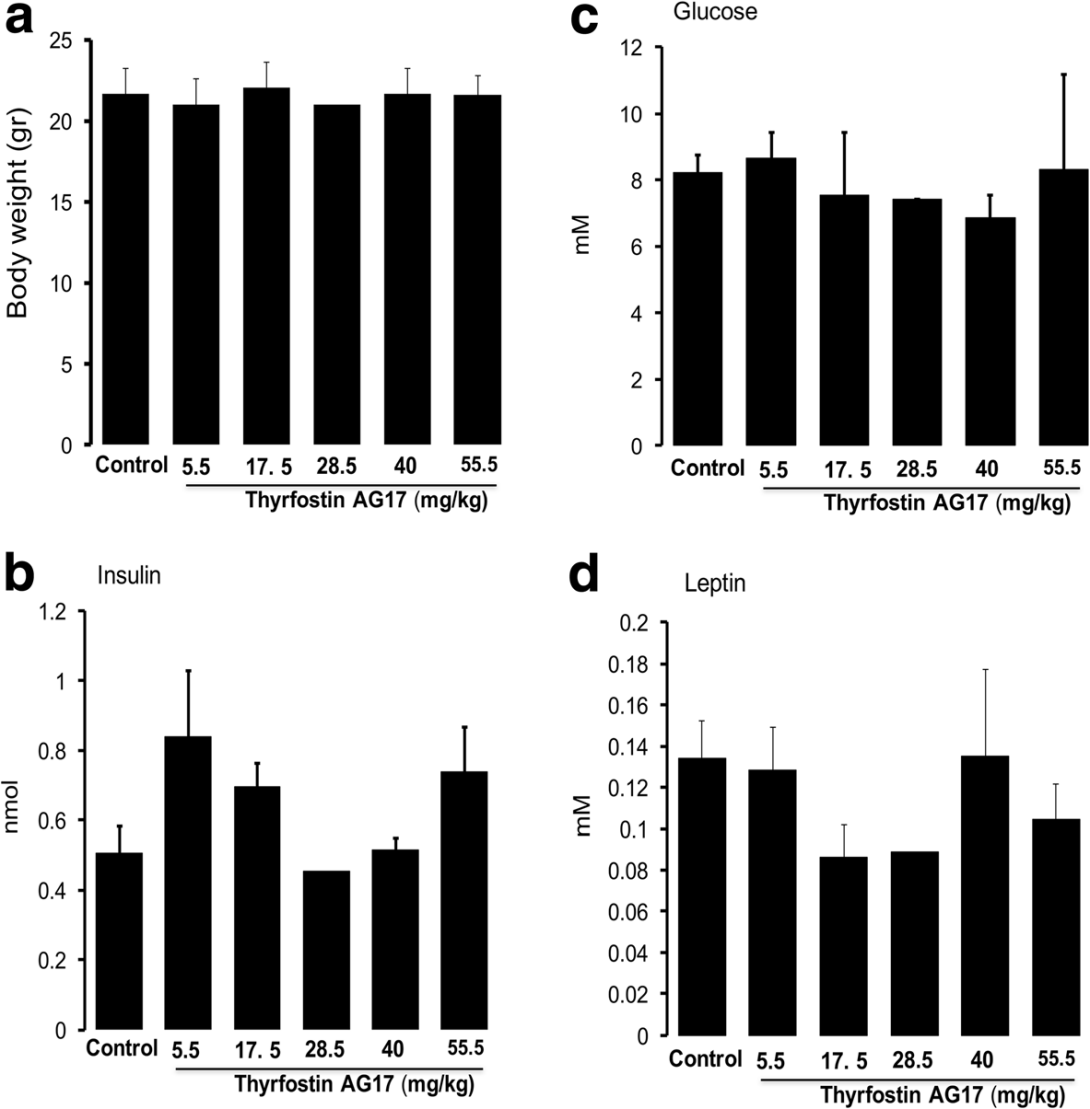
Acute T-AG17 administration does not alter body weight, glucose, insulin and leptin serum levels. **a** Body weight was analyzed every week after 17.5, 28.5, 40 and 55.5 mg/kg oral T-AG17. Changes in body weight are expressed in grams. **b**-**d** Serum biochemistry was determined using ELISA kits (insulin and leptin) as described in Methods and glucose levels measurement was determined by Glucose strips. Graphs show the normalized results of mean ± SEM for *n* = 6

We determined the LD_50_ and therapeutic dose for T-AG17 using C57BL/6 mice. We found that oral T-AG17 administration caused increased mortality at 28, 40 and 55.5 mg/kg doses, with no effect observed for 1.75, 5.5 and 17.5 mg/kg doses. The 55 mg/kg dose killed all animals within 15 min due to cardiac failure. Administration of 28 mg/kg or 40 mg/kg doses caused all animals to die after seven and 2 days, respectively. We found that 40 mg/kg administration induced a 60% mortality rate, suggesting that the LD_50_ value for this compound in mice is 33.3 mg/kg. Animals that survived after 2 weeks post T-AG17 administration (5.5, 17.5, 28.5, 40 or 55 mg/kg) did not show altered serum levels of glucose, insulin and leptin (Fig. 2).

When we looked at behavioral alterations, we observed that the animals administered with 1.75, 5.5 and 17.5 mg/kg exhibited inhibition in locomotion and piloerection, which lasted up to 30 min after administration. With 28 mg/kg, mice also exhibited increased heart rate 5 min after administration and a red appearance of the footpads. One mouse developed prostration and lung spasms after receiving 28 mg/kg. This mouse was sacrificed by cervical dislocation. Thirty minutes after T-AG17 administration, mice recovered mobility. At 40 mg/kg, mice showed the same physiological parameters as the 28 mg/kg dose, and the mice also exhibited hyperventilation Three out of five mice were sacrificed by cervical dislocation due to irreversible negative physiological effects. Two of the mice recovered after 30 min. Finally, the 55 mg/kg T-AG17 dose showed exacerbation of the behavioral and physiological parameters exhibited at the 40 mg/kg dose. The mice of this group were sacrificed between 5 and 10 min after administration.

We identify changes in cell morphology of brain, kidney, liver, pancreas, adipose tissue and muscle after 17.5, 28 and 55.5 mg/kg T-AG17 oral administration. The brain showed hyperchromic cytoplasm, perinuclear eosinophilic inclusions and dispersed chromatin. The kidneys of these mice showed pale aspects of the glomerular units and proximal tubules. The liver displayed hepatic acinar cells with perinuclear cytoplasmic vacuoles and and the acinar unit randomly distributed. The pancreas displayed exocrine acinar cells with pale appearance and normal Langerhans islets. White adipose tissue exhibited adipocytes with normal aspect and an apparent decrease in adipocyte cell number intercalated with major adipocytes evidenced by the red oil stained. The skeletal muscle of these mice seemed morphologically unaltered.

Mice orally administered 41 mg/kg T-AG17 showed neurons displaying eosinophilic and pale cytoplasm and pycnotic nucleus coexisting with normal neurons. Kidney cells seem retracted with pale glomeruli and tubular cells with poor staining, pycnotic nuclei, and condensed chromatin. Hepatocytes from the liver displayed pale cytoplasms and large perinuclear vacuoles. Some areas had low affinity for staining and others areas had disorganized hepatocytes, edema, and the appearance of fat accumulation as evidenced by oil red staining.

### Chronic doses of T-AG17 administration

**Fig. 3:**
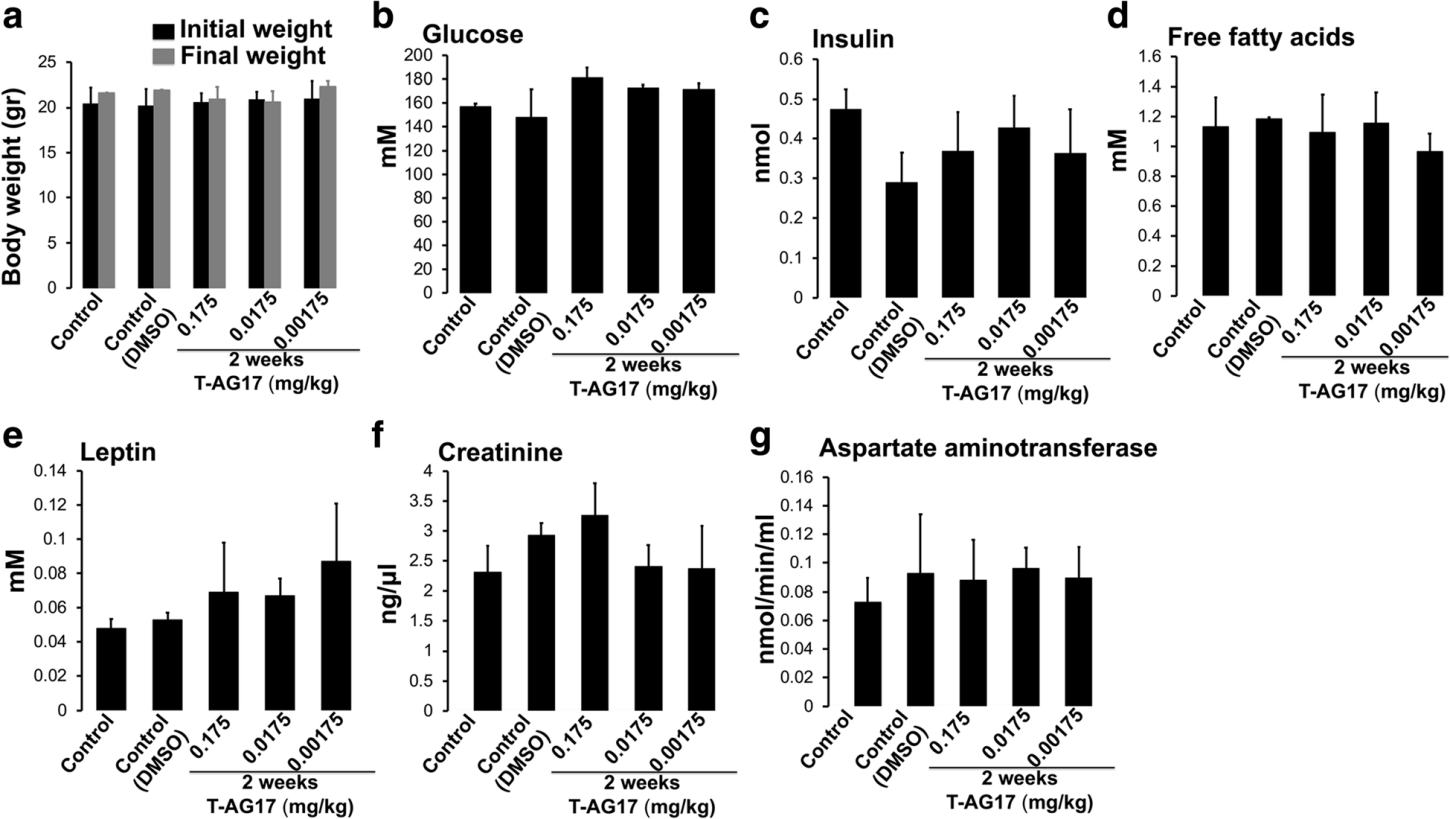
Chronic T-AG17 does not alter body weight, glucose, insulin, and leptin levels and creatinine and aspartate aminotransferase activity in serum. **a** Body weight was analyzed every week after 2 weeks of 0.0175, 0.175 and 1.75 mg/kg oral T-AG17 administration. Changes in body weight are expressed in grams. **b**-**e** Blood glucose levels were determined using glucose strips and serum biochemistry was determined using ELISA kits as described in Methods. **f**-**g** Creatinine and aspartate aminotransferase activity were determined using ELISA kits. Graphs show the normalized results of mean ± SEM for *n* = 6

In order to test the potential of T-AG17 oral administration to regulate body weight, we selected the lowest dose administered in our previous experiment (Fig. 2, oral dose AG17 = 1.75 mg/kg). Mice were orally dosed with 0.175, 0.0175 or 0.00175 mg/kg T-AG17 during 2 weeks reaching 2.45, 0.245 and 0.0245 mg/kg final concentrations after treatment (Fig. 3). We determined plasma biochemistry and tested for renal and liver damage using selective markers. We found that oral 0.175, 0.0175 or 0.00175 mg/kg T-AG17 doses did not alter plasma biochemistry markers including glucose, leptin, insulin and free fatty acids and nor the creatinine and aspartate aminotransferase (two markers of renal and liver damage, respectively) (Fig. 3).

### T-AG17 promotes apoptotic cell death of white adipocytes in obese mice

**Fig. 4:**
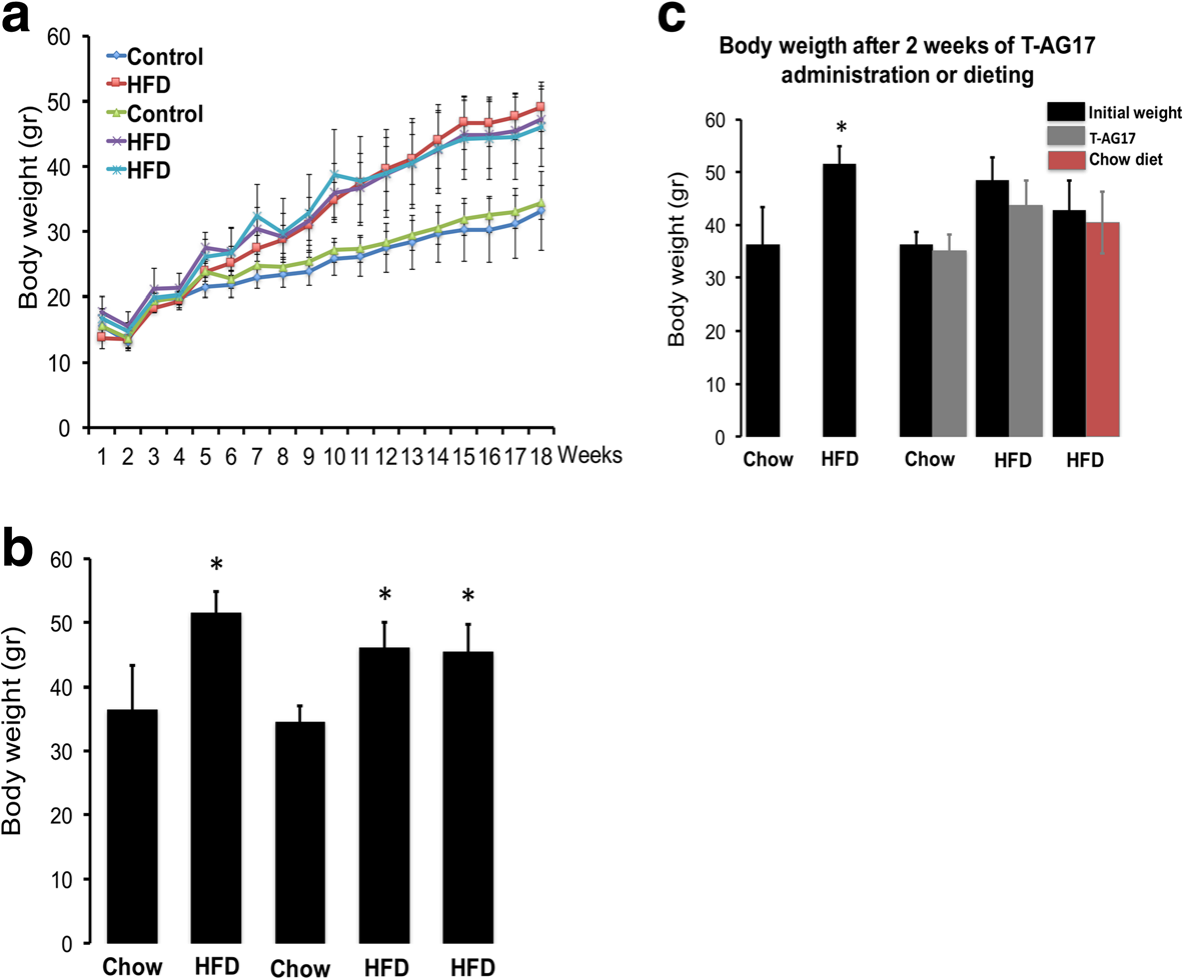
T-AG17 administration and body weight in animals exposed to HFD. **a** Mice were exposed to HFD (60% kcal from fat) or chow diet during 18 weeks. **b** Body weight was determined every week. Changes in body weight are expressed in gr. **c** Body weight change after 2 weeks of oral T-AG17 administration. Graphs show the normalized results of mean ± SEM for *n* = 9–12 and statistical significance after using unpaired Student’s t test. *p < 0.05

Based on the observation that T-AG17 stimulation promotes apoptotic cell death in 3T3-L1 cell line, we sought to determine if administration of T-AG17 in mice modulates thermogenesis or white adipose tissue apoptosis, leading to body weight decrease. Initially, we fed mice with Chow or HFD for 18 weeks and tested the potential of T-AG17 versus dieting. In this experiment, we injected T-AG17 intraperitoneally (grey bar Fig. 4c) or exposing the HFD mice to a normal Chow diet (red bar Fig. 4c) for 2 weeks. As expected, we found that 18 weeks of HFD intake increased the body weight reaching 45–50 g when compared to Chow diet values (25–28 g) (Fig. 4a, b). No significant changes in food intake were found (Chow = 23.91 ±2.07 g, HFD = 26.83 ±9.72). HFD intake increased the body weight (black bar vs Chow diet, Fig. 4c); however, we did not find evidence of weight loss after T-AG17 administration (compare black bar HFD groups vs grey bar Fig. 4c). The latter observation correlates which we found when mice were put back to the Chow diet during 2 weeks (compare black bar HFD groups vs red bar Fig. 4c).

**Fig. 5:**
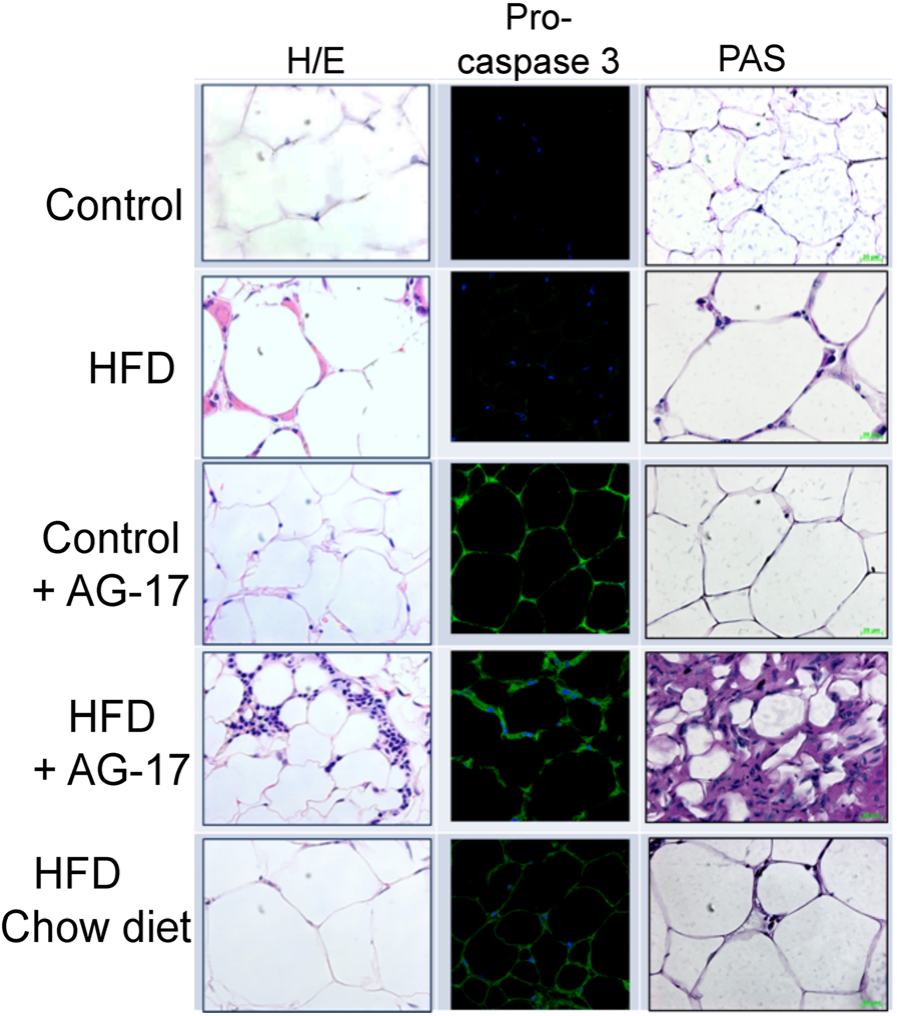
T-AG17 promotes apoptotic cell death in adipose tissue. Mice were exposed to HFD (60% kcal from fat) or Chow diet during 18 weeks and T-AG17(0.175 mg/kg) was orally administered during 2 weeks. Pro caspase 3 activation was evaluated using immunohistochemistry. PAS staining was performed to evaluate glycogen synthesis

Next, we assessed any morphological changes in WATafter T-AG17 or dieting exposure. WAT of mice fed the Chow diet showed typical polyhedral cells with nuclear location near the plasma membrane; whereas, cells of mice exposed to HFD reached a higher apparent size (Fig. 5). Notably, obese mice administered the T-AG17 showed an apparent decrease in adipocyte fat droplet size when compared to Chow and HFD groups (Fig. 5). Also, T-AG17 administration of mice expose to Chow diet did not show morphological changes when compare to Chow and HFD groups. In addition, we did not identify positive staining using the PAS protocol for visceral fat tissue in all groups (Fig. 5). T-AG17 induced a significant increase in active pro-caspase 3-Cy3 staining in obese mice when compared to controls fed with Chow diet (Fig. 5). This effect was replicated in obese mice put back to normal Chow diet for 2 weeks (Fig. 5); however, positive pro-caspase 3-Cy3 staining of cells was not significant when compared to obese mice administered T-AG17 (Fig. 5).

### T-AG17 promotes changes in hepatocytes of obese mice

**Fig. 6:**
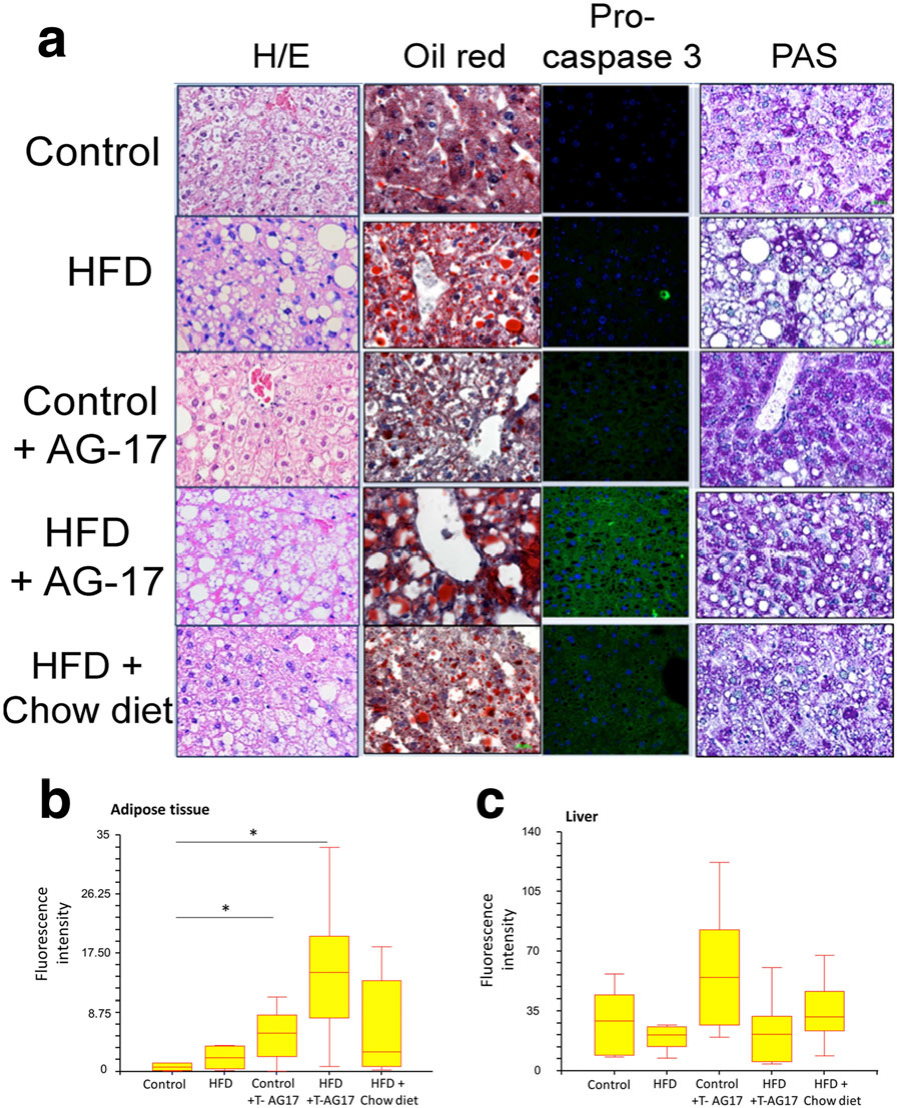
T-AG17 shifts macrovesicular to microvesicular steatosis in liver. **a** Mice were exposed to HFD or Chow as described and T-AG17(0.175 mg/kg) was orally administered during two weeks. H/E, oil red staining, pro caspase 3 activation and PAS staining were performed in adipose tissue (**b**) or liver (**c**) to evaluate morphology, fat accumulation, apoptosis activation and glycogen synthesis, respectively. For immunohistochemistry analysis, we used ANOVA test followed by Kruskal-Wallis test one-way. The data is presented as mean±SEM unless stated.. *p < 0.05

We then assessed any morphological changes in the livers after T-AG17 or dieting exposure. The livers of mice exposed to HFD showed abnormal retention of lipids displaying macrovesicular steatosis (Fig. 6a). Oral T-AG17 administration led to a transition from macrovesicular to microvesicular steatosis, which correlated with an increase in red oil positive inclusions (lipids) (Fig. 6a, b). Obese mice put back on Chow diet for 2 weeks showed a similar hepatocyte morphology as the Chow diet group and also showed a switch from macrovesicular to microvesicular steatosis. Notably, oral T-AG17 administration promoted a significant increase in caspase-3 activation in adipose tissue but not in liver when compared to HFD or Chow diet (Fig. 6b, c). Finally, obese and normal mice orally administered T-AG17 showed liver positive PAS staining (Fig. 6a), reflecting an increase of cytoplasmic glycogen inclusions.

## Discussion

We have shown that orally administered T-AG17 modulates adipocyte differentiation in vitro and induces adipocyte apoptosis in vivo. Oral administration of T-AG17 (0.175 mg/kg daily over 2 weeks) decreased fat cell volume in WAT.

Incubation with T-AG17 prevents differentiation of 3T3-L1 cells to mature adipocytes, as shown by a decrease in fat droplet formation by oil red staining (Fig. 1a, b). Fat droplet formation is one of the final stages of adipocyte differentiation [43]. This finding is consistent with the previous observations that T-AG17 suppresses insulin-mediated fatty acid synthesis in rat white adipocytes [33] and also that Tyrphostin AG490 (which bears the same pharmacophore as T-AG17) inhibits adipogenesis by disrupting STAT3 signaling in human adipocytes through inhibition of JAK2 [44]. This finding suggests that T-AG17 might be a potential effective adipogenesis-blocking agent; therefore, we tested the tolerance and therapeutic effects in a murine model of obesity.

In diet-induced obese C57BL/6 mice, T-AG17 does not induce weight loss, as we had originally hypothesized. Interestingly, the DNP uncoupler does induce weight loss at thermoneutrality (30 °C) [10], which differs from our study. However, T-AG17 did promote an apparent decrease in the size of adipocytes and a significant activation of caspase 3-dependent apoptosis (Figs. 5, 6). The decrease in adipocyte size might be mediated by MMU-induced lipolysis and down regulation of lipid synthesis [6]. Tyrphostins, in particular, have been characterized as classical inductors of apoptosis by inhibiting protein tyrosine kinase activity [30], suggesting that T-AG17 might be a potential inductor of apoptotic cell death in adipocytes. Indeed, T-AG17 has been shown to inhibit cell growth and induce apoptosis in other cell types [31, 38, 39]. These evidence suggest that T-AG17 activates the apoptotic pathway in adipocytes, although no overall weight loss was observed.

Defects in adipocyte tissue expandability and hypertrophy lead to ectopic fat accumulation in metabolically relevant organs, including liver [7]. In a fatty liver, triglyceride (TG) accumulation, either as small or large lipid deposits, is called micro- or macrovesicular steatosis, respectively [45–47]. Microvesicular steatosis, gradually followed by macrovesicular steatosis, is experimentally induced by 6–10 weeks of HFD feeding [22, 48, 49], which correlates with an elevation in liver enzymes (AST and ALT) [45, 50]. Our results agree with these findings, with mice exhibiting macrovesicular steatosis after 12 weeks of HFD feeding. To the best of our knowledge, the T-AG17- induced switch from macrovesicular to microvesicular liver steatosis in the HFD group (Fig. 6) is unprecedented. Also, while T-AG17 increases lipid inclusions in hepatocytes as shown by oil red staining (Fig. 6), we did not find any changes in serum AST levels, suggesting no histological lesions in liver. We hypothesize that this is a transient condition whereby fatty acids are being temporarily exported to the liver from WAT. In any case, our observations parallel those of Dianzani and Scuro [51], who reported increases in hepatic fat droplets in albino rats 48–96 h after injections with DNP, following by glycogen infiltration after 120 h and decreased in neutral fat droplets. T-AG17 administration for 2 weeks results in a microvesicular steatosis phenotype with normal levels of liver enzymes. Livers from human donors with moderate and severe macrovesicular steatosis are considered unfit for transplantation [53]. Therefore, our findings support a role of T-AG17 as a MMU capable of inducing the transition from macrovesicular to microvesicular steatosis in liver and potentially improving the metabolic body profile.

Finally, our findings show that obese mice treated with T-AG17 accumulate glycogen in the liver. Previously, injection of DNP in albino rats has been shown to also cause hepatic glycogen infiltration [51], and treatment of obese rats with a controlled-release formulation of DNP led to an 80% increase in liver glycogen content [18], which is associated with reversal of hypoglycemia. Our data are consistent with evidence showing that glycogen accumulates in the liver of rats following prolonged administration of DNP, in contrast to other parenchymal organs [18], and also with the finding that MMU-triggered by FCCP induces glucose uptake in adipocytes [6].

## Conclusions

T-AG17 blocks adipocyte differentiation in vivo and in vitro, promotes efficient apoptotic cell death of adipocytes in vivo, and a switch from macrovesicular to microvesicular steatosis during positive energy balance in a diet-induced obesity mice model. Thus, our data support the development of T-AG17 as a candidate to pharmacologic prevention/treatment of obesity and fatty liver disease.
